# Prediction of Chemotherapy Response of Osteosarcoma Using Baseline ^18^F-FDG Textural Features Machine Learning Approaches with PCA

**DOI:** 10.1155/2019/3515080

**Published:** 2019-07-24

**Authors:** Su Young Jeong, Wook Kim, Byung Hyun Byun, Chang-Bae Kong, Won Seok Song, Ilhan Lim, Sang Moo Lim, Sang-Keun Woo

**Affiliations:** ^1^Samsung Sotong Clinic, Namyangju, Kyeonggi-do, Republic of Korea; ^2^Division of RI-Convergence Research, Korea Institute of Radiological and Medical Sciences, Seoul 01812, Republic of Korea; ^3^Department of Nuclear Medicine, Korea Institute of Radiological and Medical Sciences, Seoul 01812, Republic of Korea; ^4^Department of Orthopedic Surgery, Korea Institute of Radiological and Medical Sciences, Seoul 01812, Republic of Korea

## Abstract

**Purpose:**

Patients with high-grade osteosarcoma undergo several chemotherapy cycles before surgical intervention. Response to chemotherapy, however, is affected by intratumor heterogeneity. In this study, we assessed the ability of a machine learning approach using baseline ^18^F-fluorodeoxyglucose (^18^F-FDG) positron emitted tomography (PET) textural features to predict response to chemotherapy in osteosarcoma patients.

**Materials and Methods:**

This study included 70 osteosarcoma patients who received neoadjuvant chemotherapy. Quantitative characteristics of the tumors were evaluated by standard uptake value (SUV), total lesion glycolysis (TLG), and metabolic tumor volume (MTV). Tumor heterogeneity was evaluated using textural analysis of ^18^F-FDG PET scan images. Assessments were performed at baseline and after chemotherapy using ^18^F-FDG PET; ^18^F-FDG textural features were evaluated using the Chang-Gung Image Texture Analysis toolbox. To predict the chemotherapy response, several features were chosen using the principal component analysis (PCA) feature selection method. Machine learning was performed using linear support vector machine (SVM), random forest, and gradient boost methods. The ability to predict chemotherapy response was evaluated using the area under the receiver operating characteristic curve (AUC).

**Results:**

AUCs of the baseline ^18^F-FDG features SUVmax, TLG, MTV, 1st entropy, and gray level co-occurrence matrix entropy were 0.553, 0538, 0.536, 0.538, and 0.543, respectively. However, AUCs of the machine learning features linear SVM, random forest, and gradient boost were 0.72, 0.78, and 0.82, respectively.

**Conclusion:**

We found that a machine learning approach based on ^18^F-FDG textural features could predict the chemotherapy response using baseline PET images. This early prediction of the chemotherapy response may aid in determining treatment plans for osteosarcoma patients.

## 1. Introduction

Osteosarcoma is a malignant tumor that primarily develops in bones of patients between 5 and 25 years of age. Osteosarcoma is a type of mesenchymal tumor that frequently metastasizes to the lungs and peripheral bone. Therefore, metastatic potential is a key factor in determining the diagnosis and prognosis of osteosarcoma [1, 2]. The introduction of neoadjuvant chemotherapy (NAC) in the treatment of osteosarcoma has led to improved prognosis and enhanced patient survival. Patient prognosis after combined NAC and surgery is better than after either treatment as monotherapy [3, 4]. In general, patients with high-grade osteosarcoma have numerous cycles of NAC before surgery. However, ineffective NAC can be toxic and may increase resistance to anticancer drugs [5]. Histological assessment of response to NAC can only be performed using resected specimens; therefore, response cannot be monitored during the course of NAC.

^18^F-fluorodeoxyglucose (^18^F-FDG) positron emitted tomography (PET) scanning is used as a tool to predict prognosis and select cancer treatment, as it allows the response to be measured before anatomical changes occur during treatment [6]. Maximum standardized uptake value (SUVmax), total lesion glycolysis (TLG), and metabolic tumor volume (MTV) are analyzed by ^18^F-FDG PET scanning; these factors are typical indicators used to predict prognosis and survival in cancer patients [7]. ^18^F-FDG uptake provides quantitative information regarding metabolism and heterogeneity of the tumors [8, 9].

Characteristics of tumors include abnormal cell growth, metabolism, immunity, and metastasis due to genetic heterogeneity, which enables cells to exhibit different properties in tumor microenvironments [10]. Intratumor heterogeneity has been associated with decreased long-term survival and has been used in assessment of prognosis. Heterogeneity in medical imaging can be evaluated using quantitative analyses of global, local, and regional areas. The textural features of global areas are calculated based on the distributions of each pixel: these include the maximum, mean, and standard deviation of the SUV, as well as the skewness, kurtosis, and 1st entropy, based on histograms. Analysis of textural features in local areas is considered to reflect differences in gray levels among pixels in those areas, such as the gray level co-occurrence matrix (GLCM) [11].

In recent years, studies have been performed using textural features for evaluation of intratumor heterogeneity, as well as prediction of survival rates in pancreatic carcinoma [12], lung cancer [13], and breast cancer [14]. Notably, several researchers have reported that SUVmax, TLG, and MTV are not significantly associated with response to NAC. Similarly, the correlations between textural features and MTV are irrelevant due to increased tumor volume [15]. Quantitative analysis has advantages in the analysis of tumors of various locations and sizes; thus, it is useful for evaluation of irregular and unstructured images [16]. Studies regarding textural features have demonstrated an association between cellular proliferation, necrosis, glucose metabolism, and intratumor heterogeneity [17]. Tumor heterogeneity, proliferation, metabolism, and angiogenesis reportedly can be predicted with quantitative analysis [18].

In this study, we acquired ^18^F-FDG PET images of patients with high-grade osteosarcoma before and after NAC. We then assessed ^18^F-FDG textural features and used machine learning to predict responses to NAC.

## 2. Materials and Methods

### 2.1. Patients

This retrospective study was conducted in a cohort of 70 patients who were diagnosed with osteosarcoma based on ^18^F-FDG PET/CT scans during the period from June 2006 to May 2017. All patients had newly diagnosed histologically proven high-grade primary osteosarcoma and received NAC with a combination of methotrexate, adriamycin, and cisplatin. Clinical characteristics, including age, were obtained from medical records and the institutional tumor registry. An experienced pathologist evaluated histological responses to NAC in the resected primary tumor, using specimens obtained during surgical treatment, based on tumor necrosis (<90%, nonresponders; ≥90%, responders) [19]. Seventy osteosarcoma patients underwent binary classification based on tumor necrosis results: thirty-seven were responders (53%) and thirty-three were nonresponders (47%). This study was approved by the Institutional Review Board and performed in accordance with the ethical guidelines of our institutional clinical research committee.

### 2.2. PET/CT Imaging

PET/CT images were acquired at baseline, as well as after the first and second cycles of NAC, using a PET/CT scanner (Biograph 6 PET/CT scanner, Siemens, Malvern, PA, USA). CT imaging was performed using a 6-slice helical CT scanner with 30 mAs at 130 kVp. After the CT scan, PET scanning was performed from the base of the skull to the thigh using 3.5 min per frame in three-dimensional (3D) mode, 60 min after intravenous injection of ^18^F-FDG (7.4 MBq/kg). PET images were reconstructed using CT for attenuation correction (field of view, 680 × 680 mm^2^; voxel size, 4 × 4 × 3 mm^3^) and 3D ordered subsets expectation maximization algorithms.

### 2.3. Quantitative Analysis

Textural features were extracted from the ^18^F-FDG PET images at baseline and after NAC (Table 1). Intratumor heterogeneity was evaluated using quantitative analysis in global, local, and regional areas. Outlines of the 3D region of interest (ROI) were identified in ^18^F-FDG PET images using the region-growing algorithm. The tumor ROI was confirmed by an experienced nuclear medicine physician. Sampling for the ROI was divided into 64 gray levels, which were verified in previous studies [15]. Quantitative analysis was assessed using the Chang-Gung Image Texture Analysis toolbox (http://code.google.com/p/cgita), an open-source software package implemented in MATLAB (ver. 2012a; MathWorks Inc., Natick, MA, USA) [20]. All calculations regarding the quantitative features workflow were performed in accordance with the Image Biomarker Standardization Initiative (IBIS), and we confirm that the features comply with this guideline [21].

### 2.4. Machine Learning Approach

Machine learning and principal component analysis (PCA) were performed using the scikit-learn package. Machine learning algorithms in this study included linear support vector machine (SVM), random forest, and gradient boosting. All machine learning approaches were trained using 80% of the osteosarcoma patients' calculated textural features; the remaining 20% of the patients' textural features were used for the test dataset. We used *k*-fold cross-validation (*k* = 10) to overcome insufficient data and resolve overfitting of the data.

Linear SVM used the L2 penalty, which is standard in support vector classification. Random forest used 100 estimators and a select entropy criterion. The gradient boosting method used 100 estimators and a Friedman MSE criterion. We also performed the PCA method with machine learning. In this study, we applied the kernel PCA method, using the RBF kernel with 44 components.

Machine learning was performed with or without PCA to compare the receiver operating characteristic (ROC) area under the curve (AUC) for prediction of the response to NAC using baseline ^18^F-FDG textural features.

### 2.5. Statistical Analysis

Significant factors in evaluation of the response to NAC were assessed using the Mann–Whitney *U* test, ROC analysis, and logistic analysis. The capacities of the features for classifying responses to NAC were investigated using the Mann–Whitney *U* test. The AUC and sensitivity of response to NAC were evaluated using ROC curve analysis. The predicted accuracies of machine learning approaches with and without PCA were assessed using independent *t*-tests. Differences with *p*-values < 0.05 were considered statistically significant. All statistical analyses were performed using MedCalc software (version 18.6, MedCalc Software bvba, Ostend, Belgium).

## 3. Results

### 3.1. ^18^F-FDG PET Image of Osteosarcoma Patients

**Figure 1:**
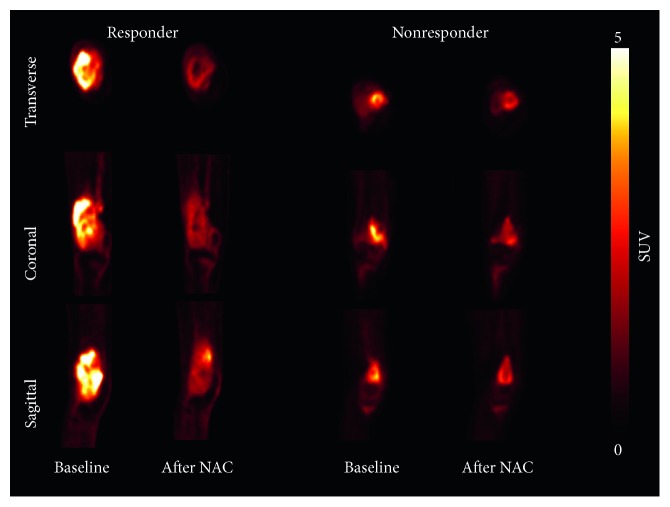
Representative ^18^F-FDG PET images of a responder and a nonresponder with osteosarcoma. Responder SUVmax values were 11.33 and 4.43 at baseline and after neoadjuvant chemotherapy (NAC), respectively. Nonresponders had SUVmax values of 5.62 and 3.21 at baseline and after NAC, respectively.

PET images in Figure 1 represent patients classified as responders or nonresponders, based on histological findings. Figure 1 depicts a responder: a 15-year-old male patient with osteosarcoma of the right femur; the SUVmax values at baseline and after NAC were 11.33 and 4.43. Figure 1 also depicts a nonresponder: an 11-year-old female patient with osteosarcoma; the SUVmax values at baseline and after NAC were 5.62 and 3.21.

### 3.2. Comparison of NAC Responder and Nonresponders

**Figure 2:**
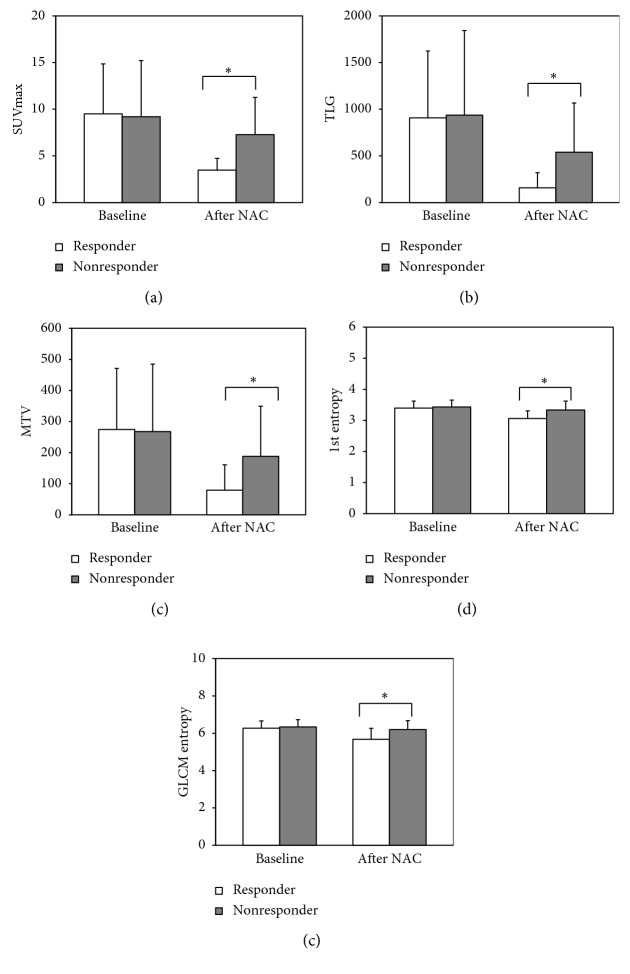
Comparison of SUVmax, TLG, MTV, 1st entropy, and GLCM entropy features value for responders and nonresponders at baseline and after neoadjuvant chemotherapy (NAC).

The Mann–Whitney *U* test was used to compare each of the PET quantitative factors between responders and nonresponders at each time point (Figure 2). SUVmax decreased by 53.3% in responders, whereas it decreased by 14.5% in nonresponders. In addition, TLG and MTV both decreased in responders and nonresponders (73.6% and 25.5%, respectively; 65.1% and 22.5%, respectively). Statistical analysis of baseline values showed that SUVmax (*p*=0.485), TLG (*p*=0.616), and MTV (*p*=0.638) did not significantly differ between responders and nonresponders. In addition, 1st entropy change decreased by 9.4% in responders and by 2.6% in nonresponders; GLCM entropy decreased by 9.1% in responders and by 2.0% in nonresponders. These differences were not statistically significant (*p*=0.616 and *p*=0.574, respectively, between responders and nonresponders). However, after NAC, all features significantly differed between responder and nonresponder groups.

### 3.3. Prediction of Response to NAC Using Textural Features AUC

**Figure 3:**
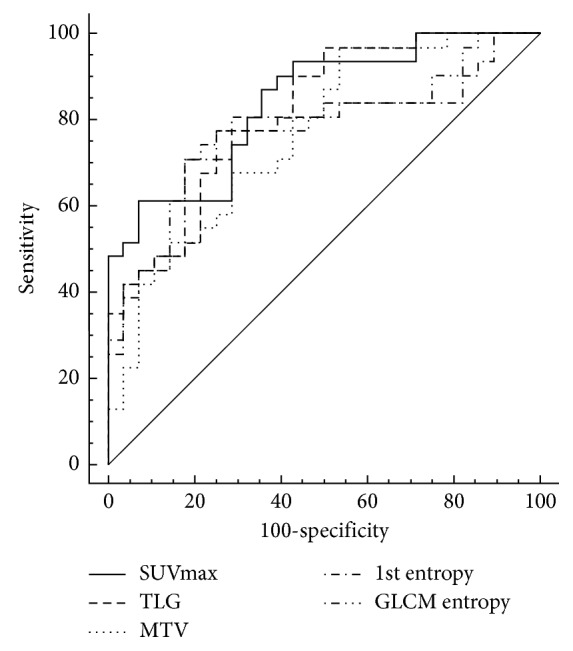
Receiver operating characteristic curves for SUVmax, TLG, MTV, 1st entropy, and GLCM entropy after neoadjuvant chemotherapy (NAC).

ROC analysis showed that baseline ^18^F-FDG PET textural features had lower AUC (SUVmax: 0.553, TLG: 0.538, MTV: 0.536, 1st entropy: 0.538, and GLCM entropy: 0.543). ROC analyses of the percent changes in textural features showed that AUCs of SUVmax, TLG, MTV, 1st entropy, and GLCM entropy were 0.863, 0.816, 0.764, 0.767, and 0.775, respectively. Notably, the AUCs of percent changes between baseline and after NAC were significantly higher than the AUCs of textural features at baseline and after NAC (Table 2).  Figure 3 shows that the ROC after NAC and all features can predict the chemotherapy response.

ROC analysis of sensitivity and specificity indicated the acceptability of the predictive model. SUVmax showed the highest sensitivity (51.61%) and 1st entropy showed the highest specificity (92.86%) in baseline ^18^F-FDG PET.

### 3.4. Prediction of Response to NAC Using Machine Learning with PCA

ROC analysis of machine learning methods without PCA showed that AUCs of linear SVM, random forest, and gradient boost were 0.54 ± 0.05, 0.58 ± 0.17, and 0.59 ± 0.12, respectively; AUCs of these methods with PCA were 0.72 ± 0.22, 0.78 ± 0.24, and 0.82 ± 0.12, respectively. The findings indicated that machine learning with PCA was superior for prediction of the response to NAC using baseline ^18^F-FDG PET data (Table 3).

## 4. Discussion

In this study, we performed quantitative analysis using ^18^F-FDG PET images of patients with high-grade osteosarcoma who underwent NAC. We used conventional factors (e.g., SUVmax, MTV, and TLG) and new quantitative factors, 1st entropy and GLCM entropy.

With the increased need for prediction of the effect of NAC cancer treatment and the corresponding survival rate, prognostic factors such as imaging histograms or textural features have gained considerable interest. In particular, textural analysis of tumor heterogeneity using local and regional characteristics has been used to predict survival in patients with pancreatic [12], lung [13], and breast cancer [14]. In a study of osteosarcoma patients, Byun et al. reported prediction of the response to NAC using global textural analysis, especially SUVmax, based on changes in PET images over 1–2.5 hours. Evaluation of the response to NAC using textural analysis has been reported in patients with esophageal cancer [18] and non-small-cell lung cancer [13]. In esophageal cancer patients, response to NAC was more accurately predicted using local features, rather than global features. Similarly, in non-small-cell lung cancer patients, local textural features (e.g., contrast, coarseness, and busyness) were superior for prediction of response to NAC [18].

Tumor heterogeneity depends on a variety of tissue components, which affect the heterogeneity of glucose metabolism [11, 16]. Moreover, increased tumor heterogeneity causes poor response to NAC [22]. In the current study, assessment of tumor heterogeneity was performed using ^18^F-FDG PET images [12, 14]. Entropy (randomness and degree of disorder was used as a representative indicator of tumor heterogeneity. Figure 2 shows that quantitative factors decreased in responder and nonresponder groups. However, none of the assessed features were significantly different in baseline ^18^F-FDG PET images. In the responder group, 1st entropy and GLCM entropy were significantly different after NAC. This result indicates that change in ^18^F-FDG uptake heterogeneity can predict the response to NAC and that 1st entropy and GLCM entropy after ^18^F-FDG PET can predict the response to NAC. The percent changes between baseline and after NAC are shown in Table 2.

The percent change of SUVmax was significantly different between responder and nonresponder groups. Furthermore, SUVmax, TLG, and MTV showed increased predictability in percent change (Table 2). ROC analysis showed that AUCs of 1st entropy and GLCM entropy were not superior to those of SUVmax, TLG, and MTV (Table 2). However, combinations of these features could predict the response to NAC using machine learning methods for analysis of baseline ^18^F-FDG PET images. Statistical result of all quantitative factors at each time point showed that at individual time points after NAC, 1st entropy and GLCM entropy were significantly different. After NAC, changes in ^18^F-FDG features SUVmax and MTV were significantly different, based on ROC analysis (*p* > 0.05). Therefore, we recommend that ^18^F-FDG PET is used to more accurately evaluate changes in ^18^F-FDG heterogeneity and predict the response to NAC in osteosarcoma patients.

ROC analysis showed that the percent changes of ^18^F-FDG textural features had higher AUC values than machine learning without PCA. These results are important for predictions in osteosarcoma patients. However, there is a disadvantage in that early treatment outcomes cannot be estimated because the percent changes of textural features require acquisition of ^18^F-FDG at baseline and after NAC. Thus, the prediction of prognosis in osteosarcoma patients using percent change of textural features is not useful, even if the prediction exhibits high accuracy. Consequently, machine learning with PCA may be more useful to predict the response to NAC in osteosarcoma patients.

Osteosarcoma is not a common cancer: fewer than 1% of all cancer diagnoses are osteosarcoma [23] and approximately 2% of childhood cancers are osteosarcoma. Therefore, it is difficult to obtain osteosarcoma ^18^F-FDG PET images and the resulting ^18^F-FDG PET data are often insufficient for robust analysis.

In this study, machine learning approaches could predict the chemotherapy response before NAC in osteosarcoma patients. However, a major limitation of this study was the insufficient size of the cohort dataset. Because osteosarcoma is an uncommon cancer, there was a restricted amount of ^18^F-FDG PET data available for our analysis. Therefore, data from a large patient cohort are needed to confirm our findings and provide a more powerful predictive model.

## 5. Conclusion

There were no significant differences between responders and nonresponders, as measured by ^18^F-FDG PET at baseline and after NAC. However, the percent change in ^18^F-FDG heterogeneity of textural features could predict the response to NAC. ROC analysis showed that the AUC of machine learning (linear SVM, random forest, and gradient boost) could predict the response to NAC using textural features in baseline ^18^F-FDG. We hope that these results help osteosarcoma patients to avoid unnecessary NAC and that they aid in selection of the appropriate treatment method for patients with osteosarcoma by predicting treatment outcomes before the initiation of NAC.

## Figures and Tables

**Table 1:** Index of textural features in global, local, and regional areas.

Feature family	Features
Intensity histogram	SUVmax
	SUVmean
	Standard deviation
	Total lesion glycolysis (TLG)
	Metabolic tumor volume (MTV)
	1st entropy

Gray level co-occurrence matrix (GLCM)	Energy
	Contrast
	Entropy
	Homogeneity
	Dissimilarity

Neighboring gray level dependence matrix (NGLDM)	Small number emphasis
	Large number emphasis
	Coarseness
	Busyness

Gray level run length matrix (GLRLM)	Short run emphasis
	Long run emphasis
	Gray level nonuniformity
	Run length nonuniformity
	Low gray level run emphasis
	High gray level run emphasis

Gray level size zone matrix (GLSZM)	Small zone emphasis
	Large zone emphasis
	Gray level nonuniformity
	Zone size nonuniformity
	Low gray level zone emphasis
	High gray level zone emphasis

**Table 2:** Receiver operating characteristic curve analysis and univariate logistic regression analysis for evaluation of response to chemotherapy.

Variable	AUC	Sen (%)	Spe (%)	*P*-value
SUVmax				
Baseline	0.553	51.61	67.86	0.488
After NAC	0.839	61.29	92.86	<0.001^*∗*^
% change	0.863	93.55	71.43	<0.001^*∗*^

TLG				
Baseline	0.538	45.16	82.14	0.626
After NAC	0.816	77.42	71.43	<0.001^*∗*^
% change	0.838	80.65	82.14	<0.001^*∗*^

MTV				
Baseline	0.536	45.16	78.57	0.645
After NAC	0.764	96.77	46.43	<0.001^*∗*^
% change	0.838	80.65	82.14	<0.001^*∗*^

1st entropy				
Baseline	0.538	22.58	92.86	0.616
After NAC	0.767	70.97	82.14	<0.001^*∗*^
% change	0.713	70.97	71.43	0.0018^*∗*^

GLCM entropy				
Baseline	0.543	41.94	75	0.575
After NAC	0.775	70.97	82.14	<0.001^*∗*^
% change	0.71	67.74	75	0.0022^*∗*^

AUC, area under the curve; Sen, sensitivity; Spe, specificity; SUV, standardized uptake value; NAC, neoadjuvant chemotherapy; TLG, total lesion glycolysis; MTV, metabolic tumor volume.

**Table 3:** Predicted mean accuracies of 10-fold validation for each machine learning approach.

Machine learning approach	Without PCA	With PCA	*P*-value
Linear SVM	0.47 ± 0.16	0.72 ± 0.22	0.0408^*∗*^
Random forest	0.62 ± 0.21	0.78 ± 0.24	0.0510
Gradient boost	0.55 ± 0.19	0.82 ± 0.12	0.0008^*∗*^

## Data Availability

The data used to support the findings of this study are available from the corresponding author upon request.
